# Reproducibility of detection of tyrosinase and MART-1 transcripts in the peripheral blood of melanoma patients: a quality control study using real-time quantitative RT-PCR

**DOI:** 10.1038/sj.bjc.6690436

**Published:** 1999-05

**Authors:** T J de Vries, A Fourkour, C J A Punt, L T F van de Locht, T Wobbes, S van den Bosch, M J M de Rooij, E J B M Mensink, D J Ruiter, G N P van Muijen

**Affiliations:** Departments of Pathology, Medical Oncology, Central Hematology Laboratory, Surgery, Medical Statistics and Dermatology, University Hospital, PO Box 9001, Nijmegen, 6500 HB, The Netherlands

**Keywords:** tyrosinase, MelanA/MART-1, circulating cancer cells, melanoma, quantitative RT-PCR

## Abstract

In recent years, large discrepancies were described in the success rate of the tyrosinase reverse transcription polymerase chain reaction (RT-PCR) for detecting melanoma cells in the peripheral blood of melanoma patients. We present a quality control study in which we analysed the reproducibility of detection of tyrosinase and MART-1 transcripts in 106 blood samples from 68 melanoma patients (mainly stages III and IV). With this study, we aimed to improve insight in the reproducibility of a RT-PCR for the detection of (minimal) amounts of circulating melanoma cells. We performed two reverse transcriptions on each mRNA sample and performed tyrosinase and MART-1 nested PCRs in duplicate per cDNA sample. Thus, four tyrosinase and four MART-1 measurements were performed per blood sample. In our study, the majority of blood samples was negative for tyrosinase (80%) or MART-1 (66%). Only four samples were positive in all four determinations for tyrosinase and seven for MART-1. Variable results (1–3 times positive results) were obtained for tyrosinase and MART-1 in 16% and 27% respectively. MART-1 PCR had a better performance than tyrosinase PCR. Sensitivity increased when both markers were used. We reasoned that the low number of melanoma marker PCR-positive blood samples can be explained by differences in mRNA quality. By using real-time quantitative PCR, we found that this was not the case: amplification of porphobilinogen deaminase (PBGD), a low copy household gene, was not different in blood samples in which a melanoma marker was not detected from groups in which this marker was detected more or less consistently (1–4 times). When applying real-time quantitative PCR for tyrosinase and MART-1, we found that a low amount of SK-MEL-28 cell equivalents was present in the blood of melanoma patients, with a higher number of equivalents in the group with a consistently positive result. We conclude that low reproducibility of a repeated assay for the detection of circulating melanoma cells is not caused by differences in mRNA quality between the samples, but due to low numbers of amplifiable target mRNA molecules in the mRNA sample. Use of more than one marker and repetition of the assay will increase the probability of finding positive PCR results. © 1999 Cancer Research Campaign


*British Journal of Cancer* (1999) **80**(5/6), 883–891
© 1999 Cancer Research Campaign
Article no. bjoc.1998.0436

### Reproducibility of detection of tyrosinase and MART-1 transcripts in the peripheral blood of melanoma patients: a quality control study using real-time quantitative RT-PCR

**TJ de Vries[1]*, A Fourkour[1], CJA Punt[2], LTF van de Locht[3], T Wobbes[4], S van den Bosch[5], MJM de Rooij[6], EJBM Mensink[3], DJ Ruiter[1] and GNP van Muijen[1]**

Departments of [1]Pathology, [2]Medical Oncology, [3]Central Hematology Laboratory, [4]Surgery, [5]Medical Statistics and [6]Dermatology, University Hospital, PO Box 9001, 6500 HB Nijmegen, The Netherlands

**Summary** In recent years, large discrepancies were described in the success rate of the tyrosinase reverse transcription polymerase chain reaction (RT-PCR) for detecting melanoma cells in the peripheral blood of melanoma patients. We present a quality control study in which we analysed the reproducibility of detection of tyrosinase and MART-1 transcripts in 106 blood samples from 68 melanoma patients (mainly stages III and IV). With this study, we aimed to improve insight in the reproducibility of a RT-PCR for the detection of (minimal) amounts of circulating melanoma cells. We performed two reverse transcriptions on each mRNA sample and performed tyrosinase and MART-1 nested PCRs in duplicate per cDNA sample. Thus, four tyrosinase and four MART-1 measurements were performed per blood sample. In our study, the majority of blood samples was negative for tyrosinase (80%) or MART-1 (66%). Only four samples were positive in all four determinations for tyrosinase and seven for MART-1. Variable results (1–3 times positive results) were obtained for tyrosinase and MART-1 in 16% and 27% respectively. MART-1 PCR had a better performance than tyrosinase PCR. Sensitivity increased when both markers were used. We reasoned that the low number of melanoma marker PCR-positive blood samples can be explained by differences in mRNA quality. By using real-time quantitative PCR, we found that this was not the case: amplification of porphobilinogen deaminase (PBGD), a low copy household gene, was not different in blood samples in which a melanoma marker was not detected from groups in which this marker was detected more or less consistently (1–4 times). When applying real-time quantitative PCR for tyrosinase and MART-1, we found that a low amount of SK-MEL-28 cell equivalents was present in the blood of melanoma patients, with a higher number of equivalents in the group with a consistently positive result. We conclude that low reproducibility of a repeated assay for the detection of circulating melanoma cells is not caused by differences in mRNA quality between the samples, but due to low numbers of amplifiable target mRNA molecules in the mRNA sample. Use of more than one marker and repetition of the assay will increase the probability of finding positive PCR results.

**Keywords:** tyrosinase; MelanA/MART-1; circulating cancer cells; melanoma; quantitative RT-PCR

The development and implementation of the extremely sensitive polymerase chain reaction (PCR) has given a great impetus to the detection of minimal loads of tumour cells in metastatic target organs or in the blood of patients (Johnson et al, 1995; Keilholz et al, 1997). For various solid tumours, mRNA of differentiation antigens can be specifically amplified by a reverse transcriptase PCR (RT-PCR). RT-PCRs have been developed for differentiation antigens such as prostate specific antigen (PSA) in prostate cancer (Brandt et al, 1996), cytokeratins (Schoenfeld et al, 1994) and carcinoembryonic antigen (CEA) (Mori et al, 1995) in epithelial cancers, and tyrosine hydroxylase in neuroblastoma (Burchill et al, 1994). Since these mRNAs are not expressed in either peripheral blood or in target organs of the metastatic process, the detection of these mRNAs is indicative for the presence of tumour cells.

A highly specific and sensitive RT-PCR detecting transcripts of the melanin synthesis enzyme tyrosinase which is specifically expressed in melanocytes and melanoma cells, was described by Smith and co-workers in 1991 (Smith et al, 1991) and has since then been used by various groups to detect melanoma cells in blood of patients with a sensitivity in the range of 1 cell of a melanoma cell line per $10^5$–$10^6$ mononuclear cells. It was soon reported that the detection of tyrosinase transcripts correlated with the stage of disease (Brossart et al, 1993; Kunter et al, 1996; Mellado et al, 1996). Also, patients with presence of melanoma cells in the blood were shown to have a shortened survival (Kunter et al, 1996; Mellado et al, 1996). A further increase in sensitivity was found when not only the conventional tyrosinase RT-PCR was performed, but also RT-PCRs for other melanoma markers on the same blood sample (Hoon et al, 1995).

Despite these paramount achievements, various pitfalls were encountered in the detection of tyrosinase transcripts by RT-PCR in the blood of melanoma patients. First, the range of tyrosinase-positive blood samples in stage IV melanoma patients varied dramatically between 0 and 100% (Brossart et al, 1993; Foss et al, 1995; Kunter et al, 1996; Pittman et al, 1996; Van der Vel-de-Zimmerman

*Received 11 August 1998*
*Revised 22 December 1998*
*Accepted 22 December 1998*

*Correspondence to:* GNP van Muijen

*Present address: Department of Cell Biology and Histology, Acedemic Medical Centre, University of Amsterdam, PO Box 22700, 1100 DE Amsterdam, The Netherlands et al, 1996; Glaser et al, 1997; Jung et al, 1997). Second, in a recent EORTC quality control study, the reproducibility of the detection of tyrosinase transcripts in blood samples which were spiked with known amounts of melanoma tumour cells was poor, with three out of seven participating study groups scoring false positive results (Keilholz et al, 1998). Third, when taking various blood samples from one patient on one day, only some of them were found positive (Reinhold et al, 1997). Fourth, results from two blood samples taken at the same time were hardly reproducible (Farthmann et al, 1998). These results indicate that RT-PCR is not the reliable technique of which highly reproducible and accurate results can be expected.

We previously reported that tyrosinase and another melanocytic lineage marker, MART-1, were heterogeneously expressed in 105 cutaneous melanocytic lesions (De Vries et al, 1997). In the present quality control study, we report in-depth on the reproducibility of the RT-PCR technique for these markers using the same RNA sample. Nested RT-PCRs for tyrosinase and MART-1 were performed four times on 106 blood samples of 68 melanoma patients. Quantitative PCRs enabled us to evaluate the quality of the mRNA samples and the quantity of tyrosinase and MART-1 transcripts.

### MATERIALS AND METHODS

### Patients

At the time of blood sampling, 106 blood samples from 68 patients with cutaneous melanoma were clinically classified according to the AJCC/UICC criteria, stages I–IV. Thus, stage I: primary melanoma ≤ 1.5 mm and/or Clark ≤ III; stage II: primary melanoma >1.5 mm and/or ≥ Clark IV and/or satellite metastases ≤2 cm from the tumour; stage III: regional lymph node metastases and/or in-transit metastases >2 cm from the primary tumour; stage IV: distant metastases. Oral consent was given by the patients after brief information on the purpose of the blood sampling. PCR outcome did not have any consequences on the treatment regime of the patients. To avoid possible contamination with cutaneous melanocytes, in each patient the first tubes were used for clinical purposes and the last tube was used for the RNA extraction and PCR analyses.

Furthermore, blood was taken from ten persons who had no history of melanoma. These ten blood samples were from healthy volunteers (four), dermatology patients with benign melanocytic lesions (two) and patients with non-melanoma malignancies (four: two patients with adenocarcinoma of the kidney, one with small cell lung carcinoma and one with testicular carcinoma).

### Blood collection and RNA isolation

Blood from melanoma patients and blood from persons with no history of melanoma was collected in 5-ml citrate tubes (Vacutainer brand blood collection tubes, Becton Dickinson, Franklin Lanes, NJ, USA). After centrifugation, the plasma was removed and replaced by a same volume of guanidinium isothiocyanate (GITC) solution containing β-mercaptoethanol to prevent RNA degradation during storage. The samples were snap-frozen in liquid nitrogen and stored at –80°C. Precautions (gloves, sterile plastic ware, DEPC-treated solutions) were taken to avoid RNAase contamination. RNA from blood samples was isolated using the GITC method (Chomczynski and Sacchi, 1987) which was previously successfully used in a similar setting (Brossart et al, 1993, 1995). A further purification was obtained with the RNAzol B method (Campro Scientific, Veenendaal, The Netherlands) following the manufacturer's instructions.

### Reverse transcription

Two micrograms of total RNA were used for RT. A mixture of RNA and 50 pmol oligodeoxynucleotide triphosphate was incubated for 5 min at 65°C and then immediately put on ice, where the other reagents were added. Altogether, the RT took place in a volume of 20-μl RT-buffer (50 mM Tris–HCl, pH 8.3) containing 75 mM potassium chloride, 3.0 mM magnesium chloride, 10 mM dithiothreitol, 200 μM of each nucleotide, 20 U RNAsin (Promega, Madison, WI, USA) and 200 U MMLV reverse transcriptase (Promega, Madison, WI, USA). After an initial incubation of 10 min at 25°C, the RT took place for 60 min at 42°C. Per incubation, 80 μl distilled water were added. From this mixture, 5 μl were used for each PCR, equal to 100 ng reverse transcribed RNA, hereby avoiding the pipetting of small volumes.

### Polymerase chain reaction

Each PCR reaction contained 75 mM Tris–HCl, pH 9.0, and 20 mM $(NH_4)_2SO_4$, 0.01% Tween (w/v), 1.0 mM magnesium chloride, 220 μM of each nucleotide and 50 pmol of each specific primer (only in the case of PBGD PCR, 30 pmol primer was used), 0.2 U Thermoperfectplus DNA polymerase (Integro, Zaandam, The Netherlands) and 0.1 μg of reverse transcribed RNA. A hot start was used, the mixture excluding the reverse primer, the dNTPs, and the DNA polymerase was heated for 5 min at 94°C, after which the remaining mixture was added per vial at 80°C. A PCR for the low copy housekeeping gene phorbobilinogen deaminase (PBGD) was included as a control for the amplifiability of cDNA. PBGD was amplified for 30 cycles of 0.5 min at 94°C followed by 1.5 min at 60°C. The tyrosinase and MART-1 PCRs were performed for 60 cycles of 0.5 min at 94°C followed by 1.5 min at 60°C followed by a nested PCR (5 μl, or 10% from the first PCR volume, was reamplified in 50 μl of a new PCR mixture) for 30 cycles at the same conditions. In the case of tyrosinase, the primers of the first PCR were HTYR1 and HTYR2, the nested reaction was performed with HTYR3 and HTYR4. These primers were used previously (Smith et al, 1991). For the detection of MART-1, we used MART1–1 and MART 1–4 as primers for the first PCR and melanA1 and melanA2 for the nested PCR (Table 1). In order to avoid contamination, no hot start preceeded the 30 cycles of nested PCR.

### Quality control aspects

In the present study, each step of the RT-PCR procedure was monitored (Table 2). Negative control blood samples were used from the RNA isolation through the whole procedure. Special precautions were taken to avoid contamination. These included: separate rooms for RNA isolation, first PCR, the nested PCR and the analysis of PCR products. Separate laboratory clothing was used in the pre- and post-PCR handling laboratories. Vials with PCR product were never opened in the pre-PCR laboratories. As a control for sensitivity of the assay, we made aliquots of RNA isolated from blood of a healthy donor which was spiked with the RNA equivalent from 1000, 500, 100, 50, 10, 5 and 1 SK-MEL-28 cells, which produces both tyrosinase and MART-1 (De Vries et al, 1997) and which has been used before in the literature to quantitate melanoma cells in the peripheral blood of melanoma patients (Brossart et al, 1995). Thus, aliquots were obtained with 1000 to 1 cell SK-MEL-28 per ml blood. Aliquots from this series were used

 © Cancer Research Campaign 1999

**Table 1** Oligomeric sequences used as primers and probes

| Target | Primers | Sequence[a] | S/AS/P[b] | Product size |
|---|---|---|---|---|
| PBGD | PBGD-5′ | 5′-CTGGTAACGGCAATGCGGCT-3′ | S | 339 bp |
| | PBGD-3′ | 5′-GCAGATGGCTCCGATGGTGA-3′ | AS | |
| | PBGD | 5′-CGAATCACTCTCATCTTTGGGCT-3′ | P | |
| Tyrosinase | HTYR1 | 5′-TTGGCAGATTGTCTGTAGCC3′ | S | 284 bp |
| | HTYR2 | 5′-AGGCATTGTGCATGCTGCTT-3′ | AS | |
| | HTYR3 | 5′-GTCTTTATGCAATGGAACGC-3′ | S | 207 bp |
| | HTYR4 | 5′-GCTATCCCAGTAAGTGGACT-3′ | AS | |
| | HTYR | 5′-TTACGGCGTAATCCTGGAAACCA-3′ | P | |
| MART-1 | MART1-1 | 5′-ATGCCAAGAGAAGATGCTCAC-3′ | S | 384 bp |
| | MART1-4 | 5′-AGCATGTCTCAGGTGTCTCG-3′ | AS | |
| | melanA1 | 5′-CACTCTTACACCACGGCTGA-3′ | S | 300 bp |
| | melanA2 | 5′-AGGTGAATAAGGTGGTGGTGA-3′ | AS | |
| | MART | 5′-CACAAGAAGGGTTTGATCATCGG-3′ | P | |

[a]From published sequences,[b]S = sense primer, AS = antisense primer, P = probe used with real-time PCR. Each probe contained a TET (tetrachloro-6-carboxy-fluorescein) reporter dye at the 5′ nucleotide and a TAMRA (6-carboxy-tetramethyl-rhodamine) quencher dye at the 3′ nucleotide.

**Table 2** Quality control aspects used in this study for the detection of melanoma marker transcripts in 106 blood samples of 68 melanoma patients

| Quality control aspect | Technical implication |
|---|---|
| Quality of mRNA | Integrity of RNA on agarose gel |
| | Amplification of low copy household gene PBGD |
| | Quantitative RT-PCR for PBGD |
| Control of contamination | Inclusion of negative control blood samples at sample preparation at each PCR round |
| | Special separation (separate rooms) for (i) RNA isolation/cDNA synthesis, (ii) PCR product analysis, (iii) laminar airflow cabinet for nested PCR |
| | Separate laboratory clothing for pre- and post-PCR handlings |
| | PCR product can never reach the sample preparation laboratory |
| Sensitivity of the assays | Nested PCR |
| | Inclusion of two melanoma markers |
| | Inclusion of a spiked blood sample with minimal amount (1 cell per ml blood) of melanoma cell line in each PCR |
| | Quantitative PCR assays for tyrosinase and MART-1 |
| Reliability of the assays | Inclusion negative controls at the final handling steps, from RNA isolation to (nested) PCR analysis |
| | Repetition (4 times) of the experiment and comparison of the results |

**Table 3** Patient characteristics according to stage

| Stage | Number of patients | Number of blood samples |
|---|---|---|
| Controls | 10 | 10 |
| Melanoma | | |
| I | 3 | 5 |
| II | 1 | 1 |
| III | 13 | 27 |
| IV | 51 | 73 |
| Melanoma total | 68 | 106 |

**Table 4** PCR results in 116 blood samples per stage of melanocytic tumour progression

| Stage | Tyrosinase + (%)[a] | MART-1 + (%)[a] | Tyrosinase and/or MART-1 + (%)[b] |
|---|---|---|---|
| Controls | 0/10(0%) | 0/10(0%) | 0/10 (0) |
| I | 2/5 (40%) | 2/5 (40%) | 2/5 (40%) |
| II | 0/1 (0%) | 0/1 (0%) | 0/1 (0%) |
| III | 4/27 (15%) | 5/27 (19%) | 8/27 (30%) |
| IV | 15/73 (21%) | 29/73 (40%) | 33/73 (45%) |

[a]A sample was called positive when at least one out of four reactions was positive. [b]At least one positive reaction out of eight determinations (four times tyrosinase, four times MART-1).

© Cancer Research Campaign 1999 

**Table 5** Reproducibility of the RT-PCRs for tyrosinase and MART-1 in 106 melanoma patient blood samples (stage I–IV)

| Number of positive reactions | Tyrosinase + | MART-1+ |
|---|---|---|
| 0 | 85 | 70 |
| 1 | 13 | 22 |
| 2 | 4 | 3 |
| 3 | 0 | 4 |
| 4 | 4 | 7 |

in each reverse transcription with the same enzyme mixture as used for the melanoma blood samples to be analysed. We deliberately titrated RNA from the cell line to RNA from blood (in stead of titrating cells to blood before the isolation), since tyrosinase and MART-1 proteins are heterogeneously expressed in this cell line (De Vries et al, 1997).

### Experimental set-up

A reverse transcription of the mRNA of each blood sample was performed twice. A total of 0.1 μg RNA (= 5 μl) was used for each PCR. From each reverse transcription mixture, at least six PCRs were performed: both tyrosinase and MART-1 in duplicate, PBGD once with the conventional PCR, and once in real-time quantitative PCR. Therefore, per blood sample, from the two RT-reactions, four tyrosinase and four MART-1 results were obtained. A blood sample which was positive for a melanoma marker in at least one out of four analyses, was called positive for that marker. Each blood sample which was positive at least once for either tyrosinase or MART-1, was analysed in the real-time quantitative PCR as well. Since the group of 106 blood samples and the ten control samples was too large to handle and analyse at once, the group was analysed in four series. In each PCR of a series, the samples spiked with melanoma cell line SK-MEL-28 served as an interexperiment control for sensitivity. Some blood samples which were negative four times in the first analysis were also analysed in real-time PCR.

### Real-time quantitative RT-PCR assay

We quantitated the PBGD housekeeping gene and tyrosinase and MART-1 transcripts by a real-time analysis (Higuchi et al, 1993; Livak et al, 1995) using the 5´ nuclease assay (Taqman$^{TM}$) (Holland et al, 1991) and the Abi Prism 7700 sequence detector (Perkin-Elmer, Foster City, CA, USA) (Gibson et al, 1996; Heid et al, 1996). In this system, PCR products are quantified by the application of a non-extendible dual-labelled probe. This probe contains at 5´ fluorogenic reporter group tetrachloro-6-carboxy-fluorescein (TET) and an internal or 3´ fluorogenic quenching group 6-carboxy-tetramethyl-rhodamine (TAMRA). Laser-induced excitation of intact probe results in fluorescent energy transfer from the reporter group to quenching group, resulting in low overall reporter group emissions. During specific amplification, hybridized probe is degraded by the nuclease activity of *Taq* polymerase. The increase in reporter group emissions is proportional to the amount of PCR product formed. Sample positivity is defined at the cycle number at which emitted fluorescence exceeds the ten times standard deviation of baseline emissions and is called the threshold cycle ($C_t$). The $C_t$ is proportional to the initial number of target molecules (Higuchi et al, 1993; Livak et al, 1995), and is used in the quantitative analysis.

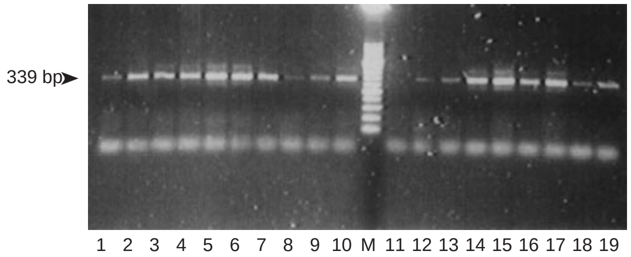

**Figure 1** Amplification of PBGD mRNA in 19 blood samples of melanoma patients. Blood samples were subjected to RNA isolation and RT-PCR with primers specific for PBGD. PCR products were subsequently visualized on an ethidium bromide stained agarose gel. Note the intensity differences between the samples. An equal amount of cDNA (100 ng) was put in each PCR reaction. M = 50-bp marker

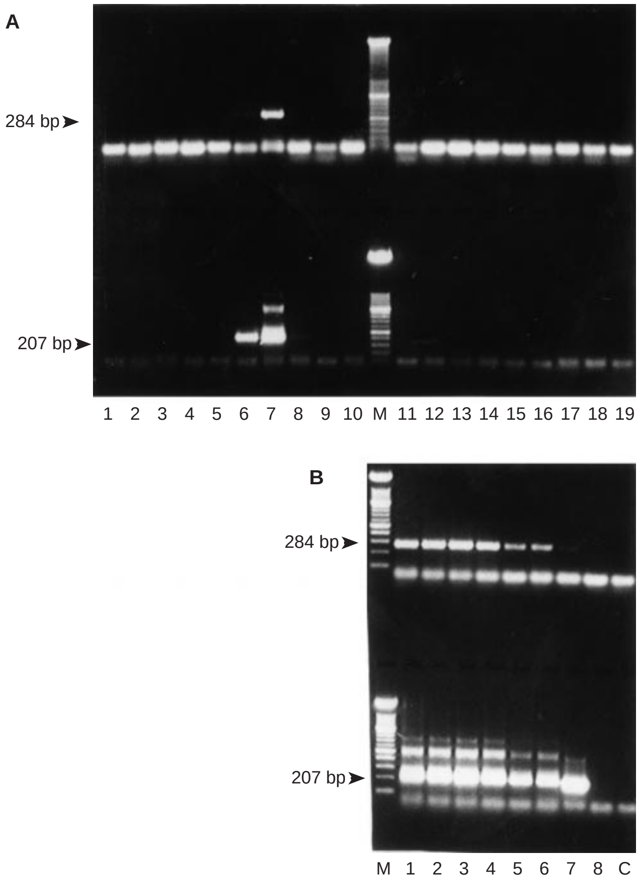

**Figure 2** Amplification of tyrosinase mRNA in 19 different patient samples and in spiked blood samples. Upper row in both panel **A** and **B** displays the first round of PCR with a specific PCR product of 284-bp, the lower half of both photographs shows the PCR product of 207-bp after nested PCR. The patient samples are shown in panel **A**. PCR results of 19 blood samples of melanoma patients are in lanes 1–19. Note that two out of 19 (both stage IV) patients show a positive PCR result after the first round (though the band in lane 6 is faint) and after the nested PCR. In panel **B**, the sensitivity test with blood spiked with the tyrosinase-positive melanoma cell line SK-MEL-28 is shown. Lane 1: 1000 cell SK-MEL-28; lane 2: 500; lane 3: 100; lane 4: 50; lane 5: 10; lane 6: 5; lane 7:1; lane 8: no SK-MEL-28 cells added. **(C)** Water control, M = 50-bp marker. Stages of patients in panel **A**: stage III (lanes 1–3, 8, 13), stage IV (lanes 4–7, 9–12, 14–19)

 *© Cancer Research Campaign 1999*

### Quantification of PBGD, tyrosinase and MART-1 transcripts

In order to compare the amplifiability of mRNA isolated from blood of melanoma patients, we performed a quantitative PCR for a low copy housekeeping gene PBGD. This test was performed in duplicate (two reverse transcriptions per mRNA sample) on each mRNA sample of the 106 melanoma patients.

The amount of tyrosinase and MART-1 transcripts present in the blood of melanoma patients could be expressed in SK-MEL-28 cell equivalents, since the dilution series with 1 to 1000 SK-MEL-28 cells per ml blood was included in each series that we tested with quantitative PCR. In each series tested, a standard curve could be made ($C_t$ versus number of cells) from the SK-MEL-28 cell equivalents. Obviously, the 1000 cell sample was detected first (lowest $C_t$), the 1 cell sample last (highest $C_t$). The 50-μl reaction mixture for each quantitative PCR contained 5 μl (0.1 μg cDNA) from the reverse transcription reaction, TaqMan buffer II (10 mM Tris–HCI pH 8.3) containing 50 mM potassium chloride, 10 mM EDTA, 60 nM ROX), 2.5 mM magnesium chloride for the PBGD PCR and 2.0 mM magnesium chloride for MART-1 and tyrosinase PCRs, 200 μM of each nucleotide, 50 pmol of each specific primer (30 pmol for PBGD primers), 10 pmol of probe (15 pmol for PBGD probe), and 1.25 U of Ampli*Taq* Gold DNA polymerase Perkin-Elmer, Foster City, CA, USA). All probes contained TET as reporter dye at the 5′ end and TAMRA as quencher dye at the 3′ end. Tyrosinase was amplified using primers HTYR 1 and HTYR4, and MART-1 was amplified using primers MelanA1 and MelanA2 (see Table 1 for sequences).

PCR conditions were the same for all targets: 10 min at 95°C (to activate *Taq* polymerase) followed by 30 at 95°C and 1 min at 60°C for 60 cycles.

### Statistical analysis

Statistical analysis was performed on the patient characteristics and test outcomes of tyrosinase and MART-1. The 106 blood samples were from 68 patients. To avoid confounding in the statistical analysis, formal statistical testing was restricted to the results of the first blood sample of each patient. Fisher's exact test was used to compare percentages of positive outcomes between stages. McNemar's test was used to compare tyrosinase and MART-1 test results.

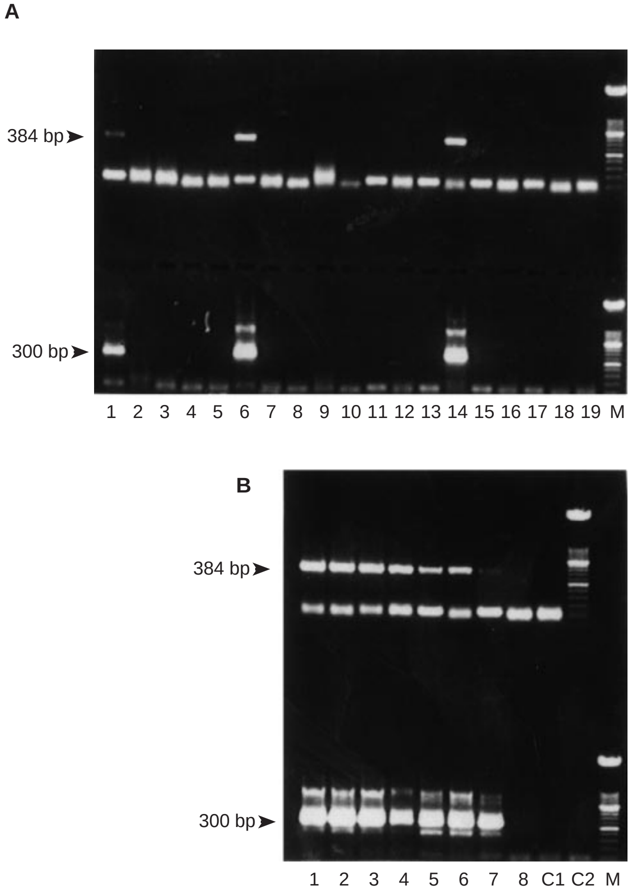

**Figure 3** Amplification of MART-1 mRNA in 19 different patient samples and in spiked blood samples. Upper row in both panel **A** and **B** displays the first round of PCR with a specific PCR product of 384 bp, the lower half of both photographs shows the PCR product of 300 bp after nested PCR. The patient samples are shown in panel **A**. PCR results of 19 blood samples of melanoma patients are in lanes 1–19. Note that three out of 19 patients (all stage IV) show a positive PCR result after the first round and after the nested PCR. In panel **B**, the sensitivity test with blood spiked with the MART-1-positive melanoma cell line SK-MEL-28 is shown. Lane 1: 1000 cell SK-MEL-28; lane 2: 500; lane 3: 100; lane 4: 50; lane 5: 10; lane 6: 5; lane 7: 1; lane 8: no SK-MEL-28 cells added. C1: water control which was included in the reverse transcription, C2: water control as a control for the nested PCR, M = 50-bp marker. Stages of patients in panel **A**: stage 0 (lanes 11 and 16), stage II (lane 3), stage III (lanes 13, 15, 18, 19), stage IV (lanes 1, 2, 4–10, 12, 14, 17)

### RESULTS

### Patient characteristics

Blood samples were collected at the University Hospital Nijmegen between November 1994 and July 1997. Initially, 115 blood samples from melanoma patients were analysed. Nine samples could not be used for this study due to poor mRNA quality or wrong anticoagulant used (heparin in stead of citrate buffer). When there was no amplification of PBGD after 40 cycles with real-time PCR (see below), the mRNA sample was excluded from the analysis.

Of the 68 patients, 41 were male and 27 female. Average age was 51.2 years, range 15.7–92.2 years. From 43 patients, one blood sample was available, from 15 patients two blood samples, from seven patients three samples, and from three patients four samples. The stage characteristics of the 68 patients and the number of blood samples used in this study are shown in Table 3. From the 25 patients with more than one blood sample, only four progressed (one patient stage II to III, three patients stage III to IV); the various blood samples of the other 21 patients were all in the same stage of disease. Our patient group contained mainly stage III and stage IV patients.

### Reproducibility of four tyrosinase and MART-1 RT-PCR determinations

A sensitive RT-PCR for the detection of a low copy household gene PBGD was used as a control for amplifiability of mRNA (Figure 1). Differences in mRNA amplifiability among the blood samples were encountered.

A nested RT-PCR for both melanocytic markers, tyrosinase and MART-1, was performed on all 106 blood samples and on ten

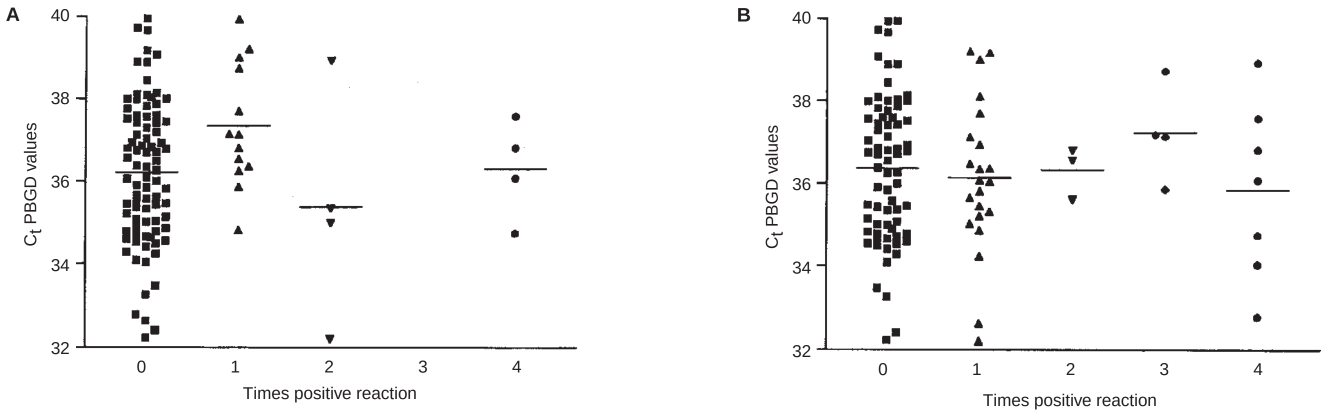

**Figure 4** $C_t$ values of PBGD PCR in blood samples which were 0–4 times positive for tyrosinase (**A**) or MART-1 (**B**). The exact number of dots per category correspond to the numbers in Table 5. No statistical differences were detected between samples which were 0 or 4 times positive for MART-1

negative control samples (non-melanoma cancer patients, healthy volunteers, dermatology patients). Both for tyrosinase and MART-1, the PCR was performed fourfold: cDNA was made in duplicate from each RNA sample, each cDNA sample was tested in duplicate for tyrosinase and MART-1. The visibility of a PCR-band after nested PCR was scored as a positive result. In Figures 2 and 3, the results for both PCRs in 19 blood samples are shown. As a quality control for sensitivity, a dilution of SK-MEL-28 (a melanoma cell line which is positive for both markers) mRNA was included in each experiment. The sample spiked with 1 cell SK-MEL-28 per ml blood was always detectable after nested PCR in each group of blood samples (for practical purposes the 106 blood samples were analysed in four groups). All ten negative controls were 80 times negative ($4 \times 10 = 40$ tyrosinase and $4 \times 10 = 40$ MART-1 determinations).

In Table 4, the results are summarized per stage of disease. In this Table, the results per blood sample were scored positive when at least one out of four reactions was positive for the melanoma marker. It can be read from Table 4 that MART-1 is more often detected than tyrosinase. When properties of positive outcomes of first blood samples (tyrosinase, MART-1) were compared with McNemar's test, MART-1 was more sensitive both in stage III and stage IV patients (McNemar's test, $P = 0.019$ and 0.039 in stage III and stage IV patients respectively). Table 4 also shows that usage of more than one marker increases the sensitivity of detection (see stage III and IV patients in the last column of Table 4). In our patient group, the finding of a positive PCR result was not associated with stage of disease for tyrosinase or MART-1 separately, but when either tyrosinase or MART-1 positivity was taken into account, the probability of at least one melanoma marker-positive outcome correlated with stage of disease (Fisher's exact test on the first blood sample of each patient, $P = 0.646$, 0.078 and 0.022 for tyrosinase, MART-1 and the combination of the two markers respectively). Since the majority of the blood samples concerned stage III and IV samples, we also tested separately for these two stages. Again, a positive test for tyrosinase or MART-1 alone did not significantly correlate with stage of disease, but the finding of at least one melanoma marker correlated positively with stage of disease (Fisher's exact test, $P = 0.432$, 0.062 and 0.028 respectively).

Since patients with stage III melanoma have a high risk for recurrence, it was of interest to examine whether RT-PCR results might be predictive of relapse. Of the 13 patients with stage III disease, seven relapsed and six remained disease-free after a median follow up of 17 months (range 10–31). Of the seven patients who subsequently relapsed, three were positive for tyrosinase/MART-1 RT-PCR at the time that stage III disease was diagnosed. All six disease-free patients tested negative for tyrosinase and MART-1. Thus, a sensitivity of 3/7 and a specificity of 6/6 was obtained in this rather small group of stage III patients.

We performed each melanoma marker PCR four times per sample. In Table 5, the reproducibility of the assay for 106 melanoma samples is presented. This Table shows that the majority of the blood samples was negative for tyrosinase (80%) or MART-1 (66%). In a reliable assay, a result should be reproducible in at least four different tests in the same sample. In our study, this was found in 89/106 (84%) when using tyrosinase as assay and in 77/106 (73%) in the case of MART-1 (Table 5). A relatively large number of blood samples was only once positive, and some samples twice or three times. From Table 5, it can be deduced that 37 ($13 \times 1 + 4 \times 2 + 0 \times 3 + 4 \times 4$) out of 424 ($106 \times 4$), or 8.7%, of the tyrosinase reactions were positive. From these 37 tyrosinase positive reactions, 27 (73%) were detectable on ethidium bromide stained agarose gel after one round of 60 cycles of PCR. Similarly, it can be deduced that 68 ($22 \times 1 + 3 \times 2 + 4 \times 3 + 7 \times 4$) out of 424, or 16.0%, of the MART-1 reactions were positive. From these 68 MART-1-positive reactions, 51 (75%) were detectable after one round of 60 cycles of PCR. Two out of seven consistently MART-1-positive samples were consistently negative for tyrosinase.

### Real-time quantification of PBGD, tyrosinase and MART-1 transcripts

The low reproducibility (number of positive reactions 1–3 in Table 4) and low percentage of positive results in stage III and IV

 © Cancer Research Campaign 1999

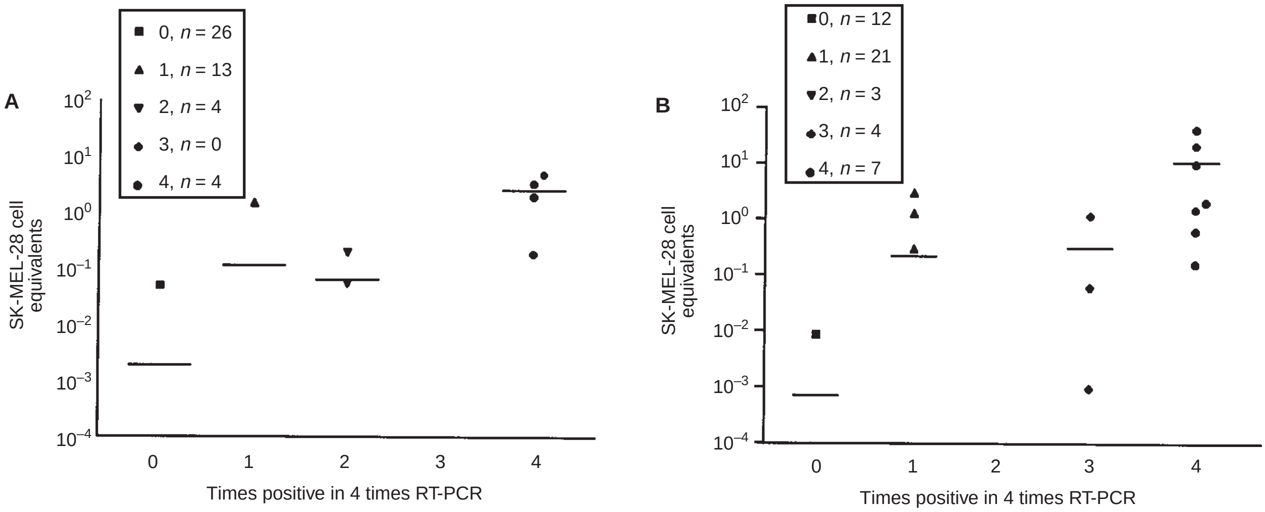

**Figure 5** Number of SK-MEL-28 cell equivalents in tyrosinase **A** and MART-1 (**B**) quantitative PCR. The results are displayed according to how often tyrosinase and MART-1 were detected in a sample in the conventional nested PCR. Forty-seven samples were tested, including all samples which were at least once positive (for corresponding numbers: see Table 5) after nested PCR (Table 5). Note that the results are displayed on a logarithmic Y-scale and that approximately half of the positive samples contained less than 1 cell patients can be explained by two factors: (a) the quality of mRNA is poor in stage III and IV patients which were four times negative compared to patients which scored four times a positive result, (b) the quality of mRNA is good, but the amount of circulating tumour cells, and therefore the number of specific target mRNA copies, is very low.

We addressed these possibilities with a real-time quantitative RT-PCR. Quantification of this PCR is based on measurements in the linear part of the amplification curve. Thus, when measuring a household gene like PBGD, the amplification in samples with good quality mRNA is first detected, at a low threshold cycle number ($C_t$). We performed the housekeeping gene PCR for all 106 blood samples twice, once for each reverse transcription reaction. Encouragingly, the $C_t$-values of the two determinations were strikingly similar. The average PBGD value of the first RT was 35.8, with a standard deviation (s.d.) of 1.8, the average PBGD value of the second RT was 36.8, with a s.d. of 2.0 Although a $C_t$ difference of 1.0 (= 1.0 PCR cycle) was found between the two RT-reactions, the 68 values (first blood sample of each patient) of the two paired measurements strongly correlated (Spearman correlation coefficient = 0.76, $P$ = 0.0001). The average of the two PBGD determinations was 36.3 (s.d. 1.8, range between 32.2 and 40.0). In Figure 4, the average of the two PBGD $C_t$ values of the 106 blood samples is plotted against the number of positive tyrosinase and MART-1 findings. We found no statistically significant differences in mRNA quality between samples which were 0, 1, 2, 3, or 4 times positive (Figure 4) (one-way ANOVA test, $P$ = 0.1560 and 0.6947 for tyrosinase and MART-1 respectively; only first blood samples were statistically tested).

Since the quality of mRNA is not significantly different between the blood samples which were consistently negative and the blood samples which were consistently positive, we then addressed the question whether the samples in which tyrosinase and MART-1 could be detected consistently contained more SK-MEL-28 cell equivalents (Figure 5). Since the majority of tyrosinase-positive (73%) and MART-1-positive (75%) samples was already detected after 60 cycles of PCR, we used 60 cycles of a quantitative PCR for tyrosinase and MART-1 to address this question. In this test, we selected 47 blood samples which included all blood samples that were positive at least once with either tyrosinase or MART-1 RT-PCR (Table 5). We also included a number of samples that were 4 times negative. A standard curve ($C_t$ vs SK-MEL-28 cell number) was made from the SK-MEL-28 cell mRNA diluted in mRNA from a healthy volunteer. As could be expected, the 1000 cell equivalent sample was detected first (thus had the lowest $C_t$ value), then the 500, 100, 50, 10, 5 and 1 cell equivalent sample respectively. All 16 standard curves (two markers, two RT-reactions, four groups, $2 \times 2 \times 4$) had a correlation coefficient above 0.95 (result not shown).

Positive measurements from the samples were expressed according to this standard curve. All negative control samples remained negative after 60 cycles of PCR, positive signals were never detected after 45 cycles. We found that very low numbers of SK-MEL-28 cell equivalents could be detected. Tyrosinase and MART-1 was detected with real-time PCR in all samples which were 4 times consistently positive for tyrosinase or MART-1 (Figure 5), whereas the number of cases which were detected only 1–3 times, were detected less often (Figure 5). It is apparent that the amount of SK-MEL-28 cell equivalents is very low: just above 1 in the case of tyrosinase and approximately 10 in the case of MART-1 in the scatter column with 4 times a positive reaction. These SK-MEL-28 cell equivalents were not corrected for differences in $C_t$ of the housekeeping gene PBGD.

### DISCUSSION

When reviewing the literature on the detection of circulating melanoma cells with use of the tyrosinase PCR, with one recent exception (Farthmann et al, 1998), no previous study has addressed the reproducibility of PCR results. Before such PCR will be applied in a clinical setting and before treatment decisions will be based on its outcome, it is of vital importance to have an understanding of how easily the PCR technique with its binary (yes or no) answers can be reproduced when using the same

*© Cancer Research Campaign 1999* 

nucleic acid preparation. In the present study we addressed this aspect of reproducibility in depth. We performed four PCRs for tyrosinase and four PCRs for MART-1 on each of the 106 blood samples from melanoma patients and found that in a considerable percentage (16% and 27% for tyrosinase and MART-1 respectively) of the 106 samples, no consistent result could be obtained (the categories 1–3 in Table 5). In a recent study, similar results were found: 33 out of 123 blood samples (27%) were 1, 2, or 3 times positive after four tyrosinase determinations (Farthmann et al, 1998). Low consistency was also found by Jung et al who repeatedly performed RT-PCRs for carcinoembryonic antigen in blood samples from patients with breast cancer (Jung et al, 1997).

In the present study, we investigated the source of this inconsistency using a quantitative PCR. We showed that the quality and amplifiability of the low copy household gene PBGD mRNA was not different in the five groups (0, 1, 2, 3, 4 times positive) for both tyrosinase and MART-1. Furthermore, the PBGD quantitative PCR taught us that PCRs are highly reproducible. This is meaningful, since a repetition of a RT-PCR using the same RNA sample inevitably means a difference in two enzyme (reverse transcriptase and TAQ polymerase) and chemical mixtures. Worrying, however, is the large range of PBGD $C_t$ values: values between 32 and 40 cycles were found. In a PCR with an efficiency of 1, this means that differences of $2^8$ or 256, arise using 100 ng RNA as measured with optical density. This means that either some RNA was degraded in the samples with a $C_t$ of 40 compared to samples with a $C_t$ of 32, or that differences of PCR inhibitory factors exist between the samples. Furthermore, since 2 μg of nucleic acid were used in each RT-reaction, differences in genomic DNA percentages in the nucleic acid solution after RNA extraction can also account for the large range of $C_t$ values. Samples with substantial amounts of DNA can give rise to lower $C_t$ values for PBGD.

With a quantitative PCR for tyrosinase and MART-1, we found very low numbers of SK-MEL-28 cell equivalents in the inconsistently (1–3 times positive) positive samples and higher number of SK-MEL-28 cell equivalents in the samples which were consistently (4 times) positive. Theoretically, as explained by Jung et al (1997), the reproducibility of a positive RT-PCR result in a sample with marker molecules present at a certain density $\lambda$ is dependent on this density. At low densities of target molecules present, the probability of reproducing the positive finding of the first determination is low. We confirmed this theoretical approach in our study using a quantitative PCR for tyrosinase and MART-1. Those samples in which a positive result was detected, zero to three tests had a lower number of SK-MEL-28 cell equivalents compared with samples in which all four tests were positive. Thus, enough target molecules should be present in a sample to allow reproducible results. In case of low densities of target molecules, the binary (yes or no) answer of the PCR will remain a matter of probability. In an earlier attempt to quantitate circulating melanoma cells according to SK-MEL-28 cell equivalents using a tyrosinase PCR and Southern blotting, similar SK-MEL-28 cell equivalents were found by Brossart et al (1995). In this study, relative quantity of tumour cells correlated with response to treatment. In addition, the same group found in this (Brossart et al, 1995) and in previous studies (Brossart et al, 1993, 1994) higher percentages of tyrosinase PCR-positive stage IV patients, incidentally reached by others (Mellado et al, 1996), but not in the majority of studies using tyrosinase PCR to detect circulating melanoma cells (Battayani et al, 1995; Foss et al, 1995; Hoon et al, 1995; Kunter et al, 1996; Pittman et al, 1996; Stevens et al, 1996; Ghossein et al, 1998; Glaser et al, 1997; Jung et al, 1997; Reinhold et al, 1997; Farthmann et al, 1998). Apart from technical differences among groups who use the tyrosinase PCR for the detection of circulating melanoma cells, differences in number of stage IV PCR-positive melanoma patients can be explained by differences in stage IV patients among the groups. It was recently found that positive tyrosinase results were less frequently observed in treated stage IV patients compared to untreated stage IV patients (U Keilholz, personal communication, manuscript in preparation). A large proportion of the stage IV patients in our study were included after a treatment regiment was applied. We previously described that tyrosinase and MART-1 are heterogeneously expressed in melanoma metastases, with approximately 17% of the metastases being negative for tyrosinase or MART-1 in immunohistochemistry (De Vries et al, 1997), this could account for some of the false negative results.

Apart from the quantitative quality control approach of PCR, another new aspect of our study is the use of MART-1 RT-PCR in the detection of melanoma cells. We show that the MART-1 PCR is more sensitive than the broadly used tyrosinase PCR in the detection of circulating melanoma cells. Furthermore, usage of more than one marker (in this study tyrosinase and MART-1), increases the sensitivity of the assay, a finding confirmed in an earlier report (Hoon et al, 1995).

Our results suggest that in stage III melanoma a positive test for melanoma cells may help to identify patients who are at risk for relapse. However, it should be noted that the number of stage III patients was small and that longer follow-up is needed. Also, a possible bias is introduced by the availability of different number of samples from stage III patients (13 patients, 27 samples).

We agree with the caution that has been advocated for the application of melanoma marker PCRs for clinical use (Buzaid and Balch, 1996; Glaser et al, 1997; Reinhold et al, 1997). Clearly, it has been shown convincingly by various groups that the likelihood of detection increases with stage of disease, but differences in sensitivity reached by the various laboratories will seriously hamper the use of melanoma marker PCR in routine clinical practice. In our view, the introduction of a stringent quality control regime using a quantitative low copy household gene PCR for the amplifiability of mRNA, amplifiability of low copy numbers of target mRNA, repetitions of positive and negative results, and introduction of multiple markers are mandatory when tests for circulating tumour cells are applied to clinical decision-making.

### ACKNOWLEDGEMENTS

We thank Jan Boezeman for helpful discussions in real-time PCR assays. This work was supported by grant number NUKC 95-912 from the Dutch Cancer Society.

### REFERENCES

Battayani Z, Grob JJ, Xerri L, Noe C, Zarour H, Houvaeneghel G, Delpero JR, Birmbaum D, Hassoun J and Bonerandi JJ (1995) Polymerase chain reaction detection of circulating melanocytes as a prognostic marker in patients with melanoma. *Arch Dermatol* **131**: 443–447

Brandt B, Junker R, Griwatz C, Heidl S, Brinkmann O, Semjonow A, Assmann G and Zanker KS (1996) Isolation of prostate-derived single cell clusters from human peripheral blood. *Cancer Res* **56**: 4556–4561

Brossart P, Keilholz U, Willhauck M, Scheibenbogen C, Mohler T and Hunstein W (1993) Hematogenous spread of malignant melanoma cells in different stages of disease. *J Invest Dermatol* **101**: 887–889

*© Cancer Research Campaign 1999*

Brossart P, Keilholz U, Scheibenbogen C, Mohler T, Willhauck M and Hunstein W (1994) Detection of residual tumor cells in patients with malignant melanoma responding to immunotherapy. *J Immunother* **15**: 38–41

Brossart P, Schmier J-W, Kruger S, Willhauck M, Scheibenbogen C, Mohler T and Keilholz U (1995) A polymerase chain reaction-based semiquantitative assessment of malignant melanoma cells in peripheral blood. *Cancer Res* **55**: 4065–4068

Burchill SA, Bradbury FM, Smith B, Lewis IJ and Selby P (1994) Neuroblastoma cell detection by reverse transcriptase-polymerase chain reaction (RT-PCR) for tyrosine hydroxylase mRNA. *Int J Cancer* **57**: 671–675

Buzaid AC and Balch CM (1996) Polymerase chain reaction for detection of melanoma in peripheral blood: too early to assess clinical value. *J Natl Cancer Inst* **88**: 569–570

Chomczynski P and Sacchi N (1987) Single-step method of RNA isolation by acid guanidinium thiocyanate-phenol-chloroform extraction. *Anal Biochem* **162**: 156–159

De Vries TJ, Fourkour A, Wobbes T, Verkroost G, Ruiter DJ and Van Muijen GNP (1997) Heterogeneous expression of immunotherapy candidate proteins gp100, MART-1 and tyrosinase in human melanoma cell lines and in human melanocytic lesions. *Cancer Res* **57**: 3223–3229

Farthmann B, Eberle J, Krasagatis K, Gstottner M, Wang N, Bisson S and Orfanos CE (1998) RT-PCR for tyrosinase-mRNA-positive cells in peripheral blood: evaluation strategy and correlation with known prognostic markers in 123 melanoma patients. *J Invest Dermatol* **110**: 263–268

Foss AJE, Guille MJ, Occleston NL, Hykin PG, Hungerford JL and Lightman S (1995) The detection of melanoma cells in peripheral blood by reverse transcription-polymerase chain reaction. *Br J Cancer* **72**: 155–159

Ghossein RA, Coit D, Brennan M, Zhang ZF, Wang Y, Bhattacharya S, Houghton A and Rosai J (1998) Prognostic significance of peripheral blood and bone marrow tyrosinase messenger RNA in malignant melanoma. *Clin Cancer Res* **4**: 419–428

Gibson UEM, Heid CA and Williams PM (1996) A novel method for real time quantitative RT-PCR. *Genome Res* **6**: 995–1001

Glaser R, Rass K, Seiter S, Hauschild A, Christophers E and Tilgen W (1997) Detection of circulating melanoma cells by specific amplification of tyrosinase complementary DNA is not a reliable tumor marker in melanoma patients: a clinical two-center study. *J Clin Oncol* **15**: 2818–2825

Heid CA, Stevens J, Livak KJ and Williams PM (1996) Real time quantitative PCR. *Genome Res* **6**: 986–994

Higuchi R, Fockler C, Dollinger G and Watson R (1993) Kinetic PCR analysis: real-time monitoring of DNA amplification reactions. *Biotechnology* **11**: 1026–1030

Holland PM, Abramson RD, Watson R and Gelfand DH (1991) Detection of specific polymerase chain reaction product by utilizing the 5′—3′ exonuclease activity of *Thermus aquaticus* DNA polymerase. *Proc Natl Acad Sci USA* **88**: 7276–7280

Hoon DS, Yuzuki D, Hayashida M and Morton DL (1995) Melanoma patients immunized with melanoma cell vaccine induce antibody responses to recombinant MAGE-1 antigen. *J Immunol* **154**: 730–737

Johnson PWM, Burchill SA and Selby PJ (1995) The molecular detection of circulating tumour cells. *Br J Cancer* **72**: 268–276

Jung FA, Buzaid AC, Ross MI, Woods KV, Lee JJ, Albitar M and Grimm EA (1997) Evaluation of tyrosinase mRNA as a tumor marker in the blood of melanoma patients. *J Clin Oncol* **15**: 2826–2831

Jung R, Ahmed-Nejad P, Wimmer M, Gerhard M, Wagener C and Neumaier M (1997) Quality management and influential factors for the detection of single metastatic cancer cells by reverse transcriptase polymerase chain reaction. *Eur J Chem Clin Biochem* **35**: 3–10

Keilholz U, Willhauck M, Scheibenbogen C, De Vries TJ and Burchill S (1997) Polymerase chain reaction detection of circulating tumour cells. *Melanoma Res* **7**: S133–S141

Keilholz U, Willhauck M, Rimoldi D, Brasseur F, Dummer W, Rass K, De Vries T, Blaheta J, Voit C, Lethe B and Burchill S (1998) Reliability of RT-PCR-based assays for detection of circulating tumour cells: a quality-assurance initiative of the EORTC melanoma cooperative group. *Eur J Cancer* **34**: 750–753

Kunter U, Buer J, Probst M, Duensing S, Dallmann I, Grosse J, Kirchner H, Schluepen EM, Volkenandt M, Ganser A and Atzpodien J (1996) Peripheral blood tyrosinase messenger RNA detection and survival in malignant melanoma. *J Natl Cancer Inst* **88**: 590–594

Livak KJ, Flood SJ, Marmaro J, Giusti W and Deetz K (1995) Oligonucleotides with fluorescent dyes at opposite ends provide a quenched probe system useful for detecting PCR product and nucleic acid hybridization. *PCR Methods Appl* **4**: 357–362

Mellado B, Colomer D, Castel T, Munoz M, Carballo E, Galan M, Mascaro JM, Vives-Corrons JL, Grau JJ and Estape J (1996) Detection of circulating neoplastic cells by reverse-transcriptase polymerase chain reaction in malignant melanoma: association with clinical stage and prognosis. *J Clin Oncol* **14**: 2091–2097

Mori M, Mimori K, Inoue H, Barnard GF, Tsuji K, Nanbara S, Ueo H and Akiyoshi T (1995) Detection of cancer micrometastases in lymph nodes by reverse transcription-polymerase chain reaction. *Cancer Res* **55**: 3417–3420

Pittman K, Burchill S, Smith B, Southgate J, Joffe J, Gore M and Selby P (1996) Reverse transcriptase-polymerase chain reaction for expression of tyrosinase to identify malignant melanoma cells in peripheral blood. *Ann Oncol* **7**: 297–301

Reinhold U, Ludtke-Handjery H-C, Schnautz S, Kreysel H-W and Abken H (1997) The analysis of tyrosinase-specific mRNA in blood samples of melanoma patients by RT-PCR is not a useful test for metastatic tumor progression. *J Invest Dermatol* **108**: 166–169

Schoenfeld A, Luqmani Y, Smith D, O’Reilley S, Shousha S, Sinnet HD and Coombes RC (1994) Detection of breast cancer micrometastases in axillary lymph nodes by using polymerase chain reaction. *Cancer Res* **54**: 2986–2990

Smith B, Selby P, Southgate J, Pittman K, Bradley C and Blair GE (1991) Detection of melanoma cells in peripheral blood by means of reverse transcriptase and polymerase chain reaction. *Lancet* **338**: 1227–1229

Stevens GL, Scheer WD and Levine EA (1996) Detection of tyrosinase mRNA from the blood of melanoma patients. *Cancer Epidemiology, Biomarkers and Prevention* **5**: 293–296

Van der Velde-Zimmerman D, Roijers JFM, Bouwens-Rombouts A, De Weger RA, De Graaf PW, Tilanus MGJ and Van den Tweel JG (1996) Molecular test for the detection of tumor cells in blood and sentinel nodes of melanoma patients. *Am J Pathol* **149**: 759–764

*© Cancer Research Campaign 1999*
